# Osteoradionecrosis of the jaws: case series treated with adjuvant low-level laser therapy and antimicrobial photodynamic therapy

**DOI:** 10.1590/1678-7757-2017-0172

**Published:** 2018-05-11

**Authors:** Guilherme Henrique Ribeiro, Mariana Comparotto Minamisako, Inês Beatriz da Silva Rath, Aira Maria Bonfim Santos, Alyne Simões, Keila Cristina Rausch Pereira, Liliane Janete Grando

**Affiliations:** 1 Universidade Federal de Santa Catarina, Núcleo de Odontologia Hospitalar do Hospital Universitário, Florianópolis, SC, Brasil.; 2 Serviço de Odontologia do Centro de Pesquisas Oncológicas de Santa Catarina, Florianópolis, SC, Brasil.; 3 Universidade de São Paulo, Departamento de Biomateriais e Biologia Oral, São Paulo, SP, Brasil.; 4 Universidade do Sul de Santa Catarina, Departamento de Odontologia, Palhoça, SC, Brasil.

**Keywords:** Osteoradionecrosis, Oral cancer, Radiotherapy, Low-level laser therapy, Antimicrobial photodynamic therapy

## Abstract

**Background:**

Osteoradionecrosis of the jaw (ORNJ) is the most severe and complex sequel of head and neck radiotherapy (RT) because of the bone involved, it may cause pain, paresthesia, foul odor, fistulae with suppuration, need for extra oral communication and pathological fracture. We treated twenty lesions of ORNJ using low-level laser therapy (LLLT) and antimicrobial photodynamic therapy (aPDT). The objective of this study was to stimulate the affected area to homeostasis and to promote the healing of the oral mucosa.

**Methods:**

We performed aPDT on the exposed bone, while LLLT was performed around the bone exposure (red spectrum) and on the affected jaw (infrared spectrum). Monitoring and clinical intervention occurred weekly or biweekly for 2 years.

**Results:**

100% of the sample presented clinical improvement, and 80% presented complete covering of the bone exposure by intact oral mucosa.

**Conclusion:**

LLLT and aPDT showed positive results as an adjuvant therapy to treat ORNJ.

## Introduction

Cancer is as a major public health problem worldwide. In 2012 14.1 million new cases were diagnosed, with 8.2 million deaths and 32.6 million people living with cancer (considering the survival time of 5 years), and 22 million new cancer cases are estimated for 2030. Oral cancer is responsible for 300.000 new cases and 145.000 deaths^9^. Thus, this disease needs attention and research for the best practices in prevention and cure.

**Figure 1:**
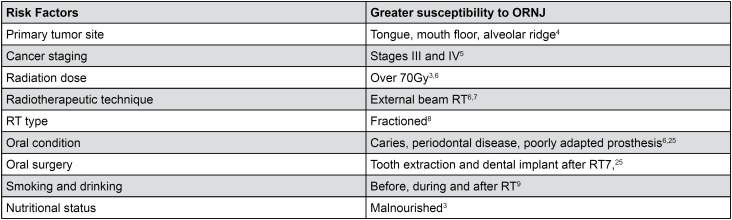
Risk factors in relation to increased susceptibility to develop ORNJ

Treatments of oral cancer include surgery, radiotherapy (RT), chemotherapy or the combination of these methods, associated or not, all of them cause sequels to the patients. The chronic side effects on the oral cavity occur mainly because of impairments on cells and/or tissues, decreasing the capacity of bone repair, especially in the jaw. Infection or trauma may cause bone necrosis^8^. The condition in which the irradiated bone is exposed to the oral cavity for at least three months is called osteoradionecrosis of the jaw (ORNJ). This condition occurs in the absence of tumor recurrence, tumor necrosis during RT or metastases in the bone^20^. Many risk factors may cause ORNJ^18^ ^,^ ^21^ ^,^ ^23^ ^-^ ^25^ ^,^ ^28^, as shown in Figure 1.

ORNJ results from imbalance in the homeostasis of all tissues affected by head and neck RT^25^. The pathophysiology is composed by three phases: (1) *initial pre-fibrotic* (injuries to endothelial cells due to reactive oxygen species caused by RT); (2) *constitutive organized* (continuous liberation of cytokines and reactive oxygen species causing abnormal fibroblastic activity); and (3) *late fibro-atrophic process* (start of a fragile tissue healing)^5^. There is no cure for established ORNJ, only clinical control, since the damage caused by head and neck RT is irreversible on the jaw^21^.

Conventional treatments to ORNJ include non-surgical debridement^20^, antibiotic therapy^4^, hyperbaric oxygenation^28^, pentoxifylline-tocopherol-clodronate combination (PENTOCLO)^26^, ultrasound^10^ and surgery^21^. Despite the numerous and well-reported therapeutic methods found in literature^7^ ^,^ ^15^ ^,^ ^17^, there are no reports of the use of low-level laser therapy (LLLT) associated with antimicrobial photodynamic therapy (aPDT).

LLLT is associated with the increase of cellular metabolism through the activation of the mitochondrial respiratory chain, increasing the levels of ATP synthesis, cell proliferation, protein synthesis and angiogenesis, which are essential for wound healing^12^. LLLT also causes an analgesic effect, by inhibiting electrophysiological activity on the nerves, altering the release of neurotransmitters that are associated with pain relief, improving lymphatic flow, among others^3^. The aPDT acts on exogenous photoreceptors, promoting the interaction of the light with a photosensitizer, producing reactive oxygen species, which causes microbial reduction^2^. Moreover, its antimicrobial action does not cause bacterial resistance or microbial selection^27^.

By reducing pain, improving the wound healing process and eliminating the opportunistic microorganisms, the use of both therapies allow the patient to continue the cancer treatment, while preserving his/her basic oral functions, such as eating, drinking, swallowing and talking. Thus, the patient is more likely to maintain a good general health status, better responding to the treatment for ORNJ. Additionally, these therapies are non-invasive, atraumatic and no significant adverse effects associated with them are reported in literature^29^.

Thus, the objective of this study was to assess the clinical effects of LLLT and aPDT to treat ORNJ in patients who underwent head and neck RT, as well as to propose an adjuvant treatment protocol to the pathology.

## Materials and methods

This is a prospective analytic experimental study. All procedures were performed at the Dentistry Center of the University Hospital of the Federal University of Santa Catarina and were in accordance with the ethical standards of the Institutional Research Committee (no. 724.398, from July/2014) and with the 1964 Helsinki Declaration and its later amendments or comparable ethical standards.

### Sample and classification of ORNJ

**Figure 2:**
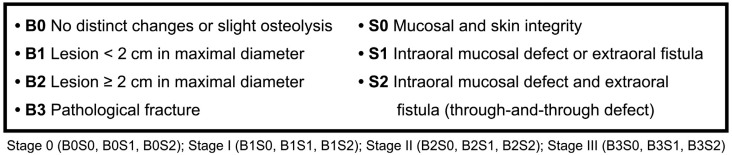
Classification of ORNJ based on clinical and imaging parameters

The inclusion criteria were patients aged over 18 years, of both sexes, who underwent head and neck RT and developed ORNJ, and who agreed with the informed consent form. The exclusion criteria were patients who developed osteonecrosis of the jaw, underage, who abandoned treatment or who did not sign the informed consent. We examined patients undergoing head and neck RT, with or without surgery and/or chemotherapy, due to cancer and primary tumor or metastasis in this area (oral cavity, oropharynx, nasopharynx and hypopharynx). The sample consisted of 20 ORNJ lesions (n=20), 6 in maxilla and 14 in mandible, which were evaluated weekly or biweekly for 2 years. The appointments depended on the availability of the patients. ORNJ lesions were graded using a clinical and imaging classification adapted from He, et al.^11^ (2015). The imaging parameters (B = bone necrosis) and the clinical parameters (S = soft tissue) are shown in Figure 2.

### LLLT and aPDT protocol for ORNJ

We used a laser device (Therapy XT^®^ - Diode laser nm, DMC, São Carlos, SP, Brazil) at λ660 nm (red spectrum) and λ808 nm (infrared spectrum), with fixed power of 100 mW. We used the red LLLT first and the aPDT after, these therapies were performed only in cases of bone exposure and/or infection. Infrared LLLT was performed imperatively, even when there was no infection or exposed bone, this procedure was performed on the interval between red LLLT and aPDT (time pre-irradiation, in which methylene blue 0.01% was used to stain the area related to bone exposure) (Table 1). The number of LLLT sessions varied for each patient, depending on the severity of the ORNJ lesion, availability of the patient for treatment and when he/she started to be treated.

**Table 1:** LLLT and aPDT protocol to ORNJ, proposed by the authors

Application site (intraoral)	LLLT (red spectrum) 100 mW, 10 s, 37.71 J/cm^2^	LLLT (infrared spectrum) 100 mW, 40 s, 142.85 J/cm^2^	aPDT (MB 0.01% 4min + red spectrum) 100 mW, 40 s, 142.85 J/cm^2^
Vestibular alveolar ridge mandible1 or maxilla2 right side	—	6 points	—
Vestibular alveolar ridge mandible1 or maxilla2 left side	—	6 points	—
Bone exposure and/or purulent secretion	—	—	1 point each 0.25 cm^2^ (0.5 cm × 0.5 cm)
Oral mucosa surrounding the bone exposure	1 point each 0.25 cm^2^ (0.5 cm × 0.5 cm)	—	—

1 From symphysis to ramus at the height of the mandibular canal; 2 From premaxilla to tuberosity at the height of the dental apexes.

### Statistical analysis

Data were analyzed using the software SPSS Statistics Version 24.0. We performed a descriptive analysis considering epidemiological features and clinical characteristics of ORNJ, and outcomes obtained through LLLT and aPDT. We performed the Chi-Square Test to verify the association between ORNJ stages and healing of the injured oral mucosa by treatment with LLLT and aPDT. The level of significance adopted was 5% (p<0.05).

## Results

The sample consisted of patients from 40 to 71 years old, the mean age was of 59.1 years. Men presented a much higher prevalence of ORNJ lesions than women, with a ratio of 9:1 cases. Only one patient had melanoderma (5%), the other patients had leukoderma (95%). When asked about drugs, most patients (85%) mentioned smoking and drinking (Table 2). There were no reports of use of illicit drugs.

**Table 2:** Epidemiological and clinical features of the sample

ORNJ lesions	Stage I	Stage II	Stage III	Total	%
**Age**					
40 – 49	0	2	0	2	10
50 – 59	0	4	2	6	30
60 – 69	5	1	3	9	45
70 – 79	3	0	0	3	15
**Sex**					
Women	0	2	0	2	10
Men	8	5	5	18	90
**Ethnicity**					
Leukoderma	8	6	5	19	95
Melanoderma	0	1	0	1	5
A**ddiction**					
Drinking and smoking	7	4	3	14	70
Smoking only	1	0	2	3	15
None	0	3	0	3	15
**Radiation dose**					
≤ 72Gy	6	4	5	15	75
> 72Gy	2	3	0	5	25
**Time elapsed between RT and ORNJ**					
≤ 24 months	6	4	0	10	50
> 24 months	2	3	5	10	50
**Site**					
Anterior maxilla	0	1	0	1	5
Posterior maxilla	2	1	2	5	25
Anterior mandible	5	2	0	7	35
Posterior mandible	1	3	3	7	35
**Extra oral communication**					
Absent	4	0	5	9	45
Present	4	7	0	11	55
**Fistula with suppuration**					
Absent	5	3	5	13	65
Present	3	4	0	7	35
**Pain**					
Absent	7	4	2	13	65
Present	1	3	3	7	35
**Paresthesia**					
Absent	5	0	3	8	40
Present	3	7	2	12	60

We could not establish a standardized RT protocol since all patients underwent head and neck RT under distinct protocols, due to being treated at different RT treatment centers.

The minimum radiation dose each patient received was 66Gy and the maximum was 92Gy, with mean total radiation dose of 72Gy. However, on five cases (25%) ORNJ was developed by receiving a total radiation dose above the mean, these patients did not reach stage III. All cases of ORNJ stage III occurred after 24 months. Half the patients presented the first ORNJ lesions within 24 months after the start of RT while the other half presented lesions after this period. Regarding where the ORNJ lesions occurred, we confirmed higher percentages on the mandible (70%). The lesions on the mandible were uniformly distributed for both the posterior (50%) and anterior (50%) regions, while the maxilla was predominantly affected on the posterior region (83.3%). The absence or presence of clinical findings such as extra oral communication, fistula with suppuration, pain and paresthesia were also assessed and are described on Table 2.

**Figure 3:**
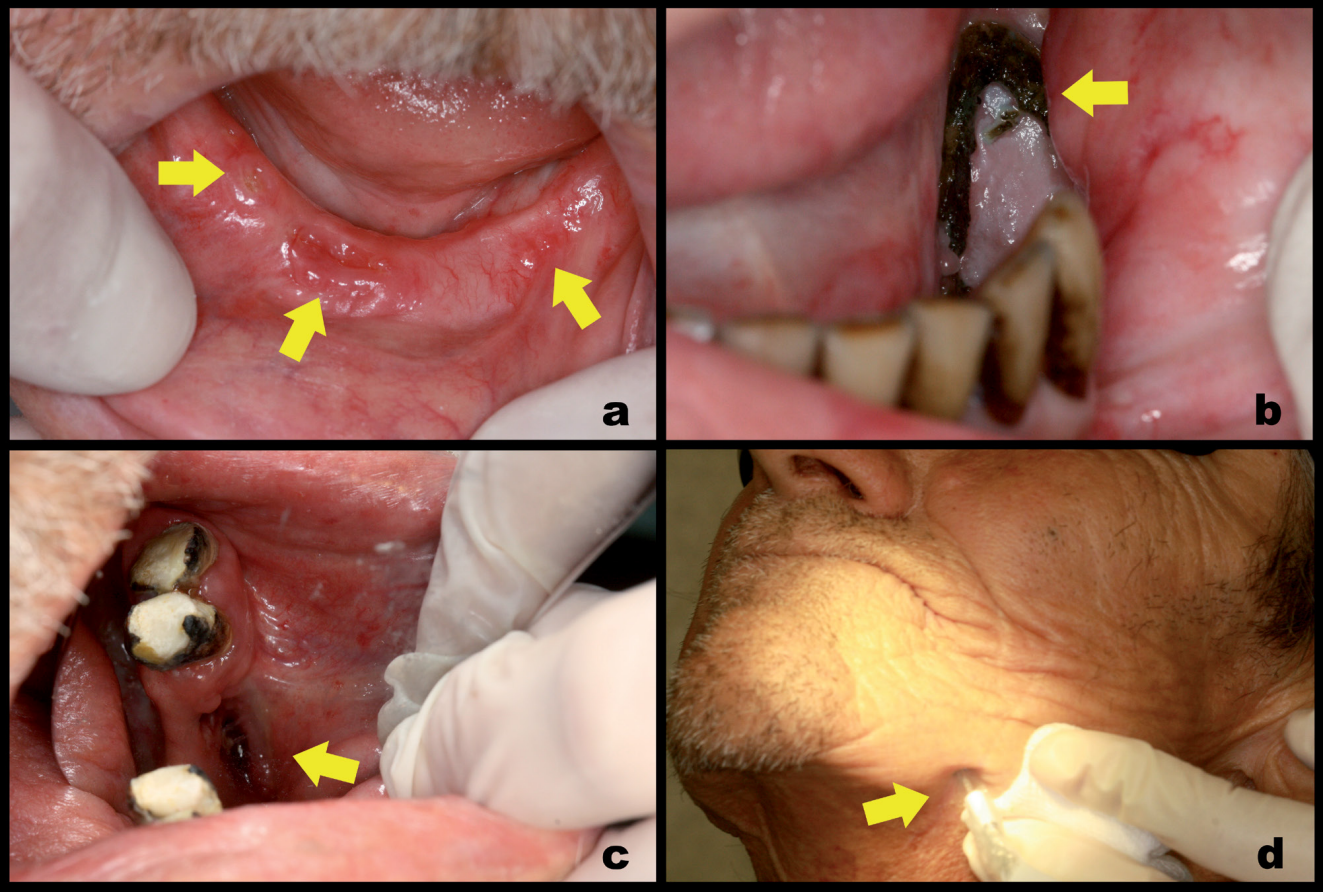
Patients with ORNJ. Stage I: bone exposures < 2 cm, asymptomatic, without evidence of infection (a). Stage II: bone exposure ≥2 cm, symptomatic, with infection, erythema and pain (b). Stage III: orocutaneous fistula with purulent drainage in intraoral (c) and extraoral (d) view

All ORNJ lesions were classified as stage I (Figure 3a), stage II (Figure 3b) or stage III (Figure 3c and 3d) to promote the most appropriate and efficient approach. No lesion was diagnosed as stage 0. Eight lesions were identified as stage I (40%), 7 as stage II (35%) and 5 as stage III (25%). After the classifying the lesions the treatment and monitoring began. Every patient was instructed on the need to maintain good oral health, hygiene of dental prostheses and regular flossing when indicated. Another standard measure was the prescription of chlorhexidine gluconate 0.12% to be used as mouth wash for 1 minute after regular oral hygiene, twice a day and continuously, regardless of the stage.

We chose some variables to analyze the therapy response to LLLT and aPDT. Stage I lesions received the most LLLT (both infrared and red spectra) sessions, while stage III received the most aPDT sessions. One of the cases of ORNJ stage I was submitted to 44 infrared spectrum LLLT sessions, 25 red spectrum LLLT sessions and 23 aPDT sessions while these numbers were 9, 11 and 21 for ORNJ stage II and 27, 16 and 38 for ORNJ stage III, respectively. The anterior mandible was the most irradiated region by LLLT and the posterior mandible was the region by aPDT. The posterior maxilla was the least irradiated region by both treatments.

Regarding the total radiation dose received during cancer treatment, the patients who underwent head and neck RT over 72Gy needed fewer sessions of LLLT and aPDT. Despite most patients receiving radiation doses lower than 72Gy, in some cases it was necessary to quadruple the infrared spectrum LLLT sessions and to triple both red spectrum LLLT and aPDT sessions to achieve better results. Similar findings were observed on the period between RT and the first record of ORNJ. Cases that presented ORNJ stages I and II within the first 24 months demanded almost twice as many LLLT sessions when compared to those who diagnosed after this period. However, a greater number of aPDT sessions were required to treat ORNJ stage III, after 24 months of the ending of RT.

LLLT and aPDT were applied multiple times during the 2 years of follow-up. In total, lesions stage I received 229 infrared spectrum LLLT, 137 red spectrum LLLT and 152 aPDT sessions. For stage II and stage III lesions we performed 83, 35 and 49 sessions and 83, 48 and 118 sessions, respectively. Considering only the cases in which there was complete remission of the ORNJ lesion, on average, it took 34.38 sessions and 17 weeks to treat ORNJ stage I, 16.17 sessions and 8 weeks to treat ORNJ stage II, and 39 sessions and 19 weeks to treat ORNJ stage III.

There was clinical improvement in 100% of the ORNJ lesions. The criteria for improvement of the clinical condition were the remission of fistulae, absence of necrosis on bone exposure, control of infections by the absence of pain, no suppuration or paresthesia, as well as the partial or total repair of the oral tissue. These effects were observed after both treatments. LLLT and aPDT were clinically effective in all cases, and in most of them (80%) the oral mucosa was fully coated (Table 3), reducing microbial contamination and avoiding the possibility of opportunistic infections through the oral cavity. The other lesions (20%) not completely healed (n=4) were classified mainly as ORNJ stage III (n=3).

**Table 3:** LLLT and aPDT clinical outcomes on ORNJ lesions

ORNJ lesions	Stage I	Stage II	Stage III	Total	%	*p*
**Clinical condition**						*.028*
Remission with partial coating of oral mucosal lining	0	1	3	4	20	
Remission with total coating of oral mucosal lining	8	6	2	16	80	

Chi-Square Test. Value with statistical significance is in italics.

## Discussion

**Figure 4:**
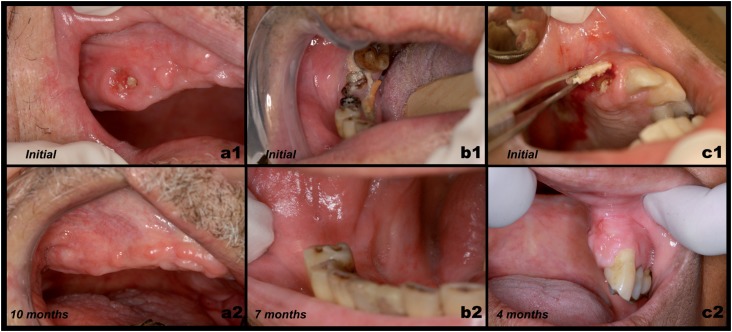
Osteoradionecrosis of the jaw (ORNJ) remission with total coating of oral mucosal lining in patients treated with LLLT and aPDT. ORNJ stage I in posterior maxilla in remission after 10 months (a). ORNJ stage II in posterior mandible in remission after 7 months (b). ORNJ stage II in anterior maxilla in remission after 4 months (c). Both patients were followed-up for two years and so far there was no recurrence of the lesions

ORNJ is a severe and complex sequel of head and neck RT that may cause deep biological, sociological and psychological impacts on the quality of life of patients. Despite ORNJ being a controllable, but not curable disease, we note that even for the most severe lesions, the results obtained with the proposed treatments exceeded our expectations (Figure 4), since the beneficial effects (absence of pain, elimination of infection and suppuration, oral mucosa repair) were observed immediately after the initial sessions. Thus, the use of LLLT, with or without aPDT, can provide excellent results to control the disease, regardless of the stage.

The results of identification and characterization of the sample for this study were similar to those already established in global literature: oral cancer is more frequent starting at 50 years old; it affects more men than women and may even exceed the ratio of 5:1 cases; leukoderma patients are more affected when compared to other ethnic groups; smoking and drinking are extremely harmful habits, which may trigger or enhance the disease^14^. According to literature, the radiation dose related to ORNJ is 70Gy^16^ ^,^ ^24^ ^,^ ^28^. Therefore, the mean radiation dose of 72Gy observed in this study was capable of inducing ORNJ, however, some patients developed ORNJ with lower doses.

Regarding the time to develop ORNJ, He, et al.^11^ (2015) reported that 68% of the patients developed ORNJ within 24 months. In contrast, Notani, et al.^19^ (2003) obtained a mean of 27.7 months, Oh, et al.^21^ (2009) 30 months and D'Souza, et al.^6^ (2007) 48 months. Despite some studies pointing means that exceed 24 months, we believe that this disease will become increasingly frequent in patients who underwent head and neck RT, which is what the most recent studies^3^ ^,^ ^13^ ^,^ ^23^ ^,^ ^25^ indicate. Several authors point the posterior mandible as the region most commonly affected by ORNJ, since the blood supplied to the mandible comes through the inferior alveolar artery, which is a facial artery and is significantly lower than the maxilla^23^ ^,^ ^24^. Furthermore, the cortical bone from the premolar to retromolar regions is considered the most vulnerable area to ORNJ. This region receives higher radiation doses during cancer treatment due to the greater bone density^20^.

The treatment for ORNJ requires a broad spectrum of processes such as patient education regarding oral hygiene and harmful habits (smoking and drinking)^23^, non-surgical debridement (sequestrectomy)^20^, mouthwash with chlorhexidine gluconate 0.12%^21^, long-term antibiotic therapy^4^, hyperbaric oxygenation^28^, ultrasound^10^, PENTOCLO combination^5^ ^,^ ^26^ and extensive surgery^6^ ^,^ ^21^. The prescription of each therapeutic modality is individualized and some of them are controversial, however, all require long periods of intervention and none of them is definitive. For these reasons patients with ORNJ must be regularly monitored due to the high risk of disease progression. Proposing the use of LLLT and aPDT to treat ORNJ is grounded on the properties of these therapies and supported by metabolism-activating effects on bone, mucosa and tissues, both very well documented in literature^2^ ^,^ ^3^ ^,^ ^12^ ^,^ ^27^ ^,^ ^29^.

The effectiveness of LLLT is supported by studies that point the effects of the therapy on the healing process of the oral mucosa by reducing the exudative phase, stimulating the healing process and by inducing the proliferation and transformation of fibroblasts and myofibroblasts^1^. The therapy also increases the blood flow by angiogenesis through revascularization and capillary growth^22^. The acceleration of the tissue repair process occurs due to the release of growth factors (TGF, PDGF, FGF-β, IL6, IL8, IL1-α) that accelerate the formation and deposition of collagen type I and III^30^ and by eliminating infections through antimicrobial aPDT action^29^.

According to the results of this study, we found that aPDT applied directly to an exposed bone with suppuration can be beneficial to control infected ORNJ lesions. Furthermore, we observed the remission of ORNJ and partial or total repair of the oral mucosa. Therefore, we can claim that LLLT and aPDT were essential to the success of disease control, reinforcing the importance of its applicability and indication.

## Conclusion

The results of this study suggest that LLLT and aPDT as a new treatment of ORNJ brought important benefits to the patients, assisting on the clinical management of the disease.

The new therapeutic approach proposed led to a decrease on the stage of ORNJ lesions, acting as an adjuvant treatment within a set of clinical maneuvers, bringing beneficial effects to control the disease and improving the quality of life of the patients. All patients in the sample benefited from the new treatment performed.

Based on our results, we recommend the use LLLT, in both red and infrared spectra, and aPDT as an adjuvant treatment of ORNJ. We suggest further research to obtain more relevant data on the action of LLLT and aPDT to treat ORNJ lesions.

## Ethics in Science

The manuscript has not been submitted to more than *Journal of Applied Oral Science* for simultaneous consideration. The material has not been published previously (partly or fully). This study is not split up into several parts to increase the quantity of submissions and submitted to various journals or to one journal over time. No data have been fabricated or manipulated (including images) to support our conclusions.

All procedures performed in this study involving human participants were in accordance with the ethical standards of the institutional and/or national research committee (n.724.398, from July/2014) and with the 1964 Helsinki Declaration and its later amendments or comparable ethical standards.

## Informed Consent

All participants of this study provided consent
